# Exposure to ambient particulate matter alters the microbial composition and induces immune changes in rat lung

**DOI:** 10.1186/s12931-017-0626-6

**Published:** 2017-07-25

**Authors:** Naijian Li, Fang He, Baoling Liao, Yuming Zhou, Bing Li, Pixin Ran

**Affiliations:** 1 State Key Laboratory of Respiratory Disease, The First Affiliated Hospital of Guangzhou Medical University, 151 Yanjiang Road, Guangzhou, 510120 China; 2 The School of Basic Medicine, Guangzhou Medical University, Guangzhou, China; 3 The GMU-GIBH Joint School of Life Sciences, Guangzhou Medical University, Guangzhou, China

**Keywords:** Particulate matter, Biomass fuel, Motor vehicle exhaust, Microbial composition, Immune response

## Abstract

**Background:**

Ambient particulate matter exposure has been shown to increase the risks of respiratory diseases. However, the role of the lung microbiome and the immune response to inhaled particulate matter are largely unexplored. We studied the influence of biomass fuel and motor vehicle exhaust particles on the lung microbiome and pulmonary immunologic homeostasis in rats.

**Methods:**

Fifty-seven Sprague–Dawley rats were randomly divided into clean air (CON), biomass fuel (BMF), and motor vehicle exhaust (MVE) groups. After a 4-week exposure, the microbial composition of the lung was assessed by 16S rRNA pyrosequencing, the structure of the lung tissue was assessed with histological analysis, the phagocytic response of alveolar macrophages to bacteria was determined by flow cytometry, and immunoglobulin concentrations were measured with commercial ELISA kits.

**Results:**

There was no significant difference in lung morphology between the groups. However, the BMF and MVE groups displayed greater bacterial abundance and diversity. *Proteobacteria* were present in higher proportions in the MVE group, and 12 bacterial families differed in their relative abundances between the three groups. In addition, particulate matter exposure significantly increased the capacity of alveolar macrophages to phagocytose bacteria and induced changes in immunoglobulin levels.

**Conclusion:**

We demonstrated that particulate matter exposure can alter the microbial composition and change the pulmonary immunologic homeostasis in the rat lung.

## Background

Particulate matter (PM) refers to an air-suspended mixture of pollutants, which contains a complex mixture of smoke, dust, and other solid and liquid materials, as well as numerous biological components. Inhaled ambient PM represents important environmental exposures that have been linked to death and disease [1, 2]. Emerging epidemiological evidence also suggests that these exposures increase the risks of respiratory diseases [3–6]. Research on PM has mostly focused on the lung inflammatory response to inhalation, as this is considered the primary impact of PM exposure [7–10]. Recent studies have linked changes in the lung microbiome to human disease, but the effects of PM on the lung microbiome are largely unknown [11, 12].

Research using culture-independent, sequence-based techniques such as 16S ribosomal RNA microarrays has shown that the lungs are not sterile [13, 14]. There is a growing understanding that the lung microbiome plays a critical role in health and disease [11]. In addition, the lung microbiome community structure may not only reflect the presence of disease but also be associated with disease features and severity [15], adding to the complexity of characterizing polymicrobial-host interactions in the lung and their potential mechanistic relationship to chronic respiratory diseases. Previous studies have found that airborne PM exposure alters the gut microbiome and induces acute and chronic inflammatory responses in the intestine [16, 17]. In spite of the increasing attention paid to the gut microbiome in PM exposures, few studies have attempted to elucidate the role of the lung microbiome in response to inhaled PM. In this study, we hypothesized that exposure to ambient PM may alter the microbial composition and induce immune changes in the lung.

## Methods

### Animals

Male Sprague–Dawley rats (7–9 weeks in age) were obtained from Guangzhou University of Chinese Medicine (Guangzhou, China). The experimental protocol and animal care complied with the guiding principles for the care and use of laboratory animals recommended by the Chinese Association for Laboratory Animal Science Policy. A total of 57 rats were randomly divided into three groups (control, biomass fuel [BMF], and motor vehicle exhaust [MVE]; *n* = 19 per group). All rats were kept in a specific pathogen-free room and were housed two to a cage. The animal facility maintained temperature control and a 12-h light/dark cycle. Rats were given standardized food and water ad libitum, and were allowed to adjust to their environment for at least 7 days before the experiments.

### PM exposure system

Rats were exposed to PM as described previously [10]. The control group was exposed to clean air every day for 4 weeks. PM mass concentrations and particle size distributions were monitored in real time during the exposure by a DustTrak II aerosol monitor 8530 (TSI, Shoreview, MN, USA). At the same time, gas concentrations (O_2_, carbon monoxide [CO], nitrogen oxides [NO_X_], and sulfur dioxide [SO_2_]) in the exposure rooms were monitored by a Testo 340 portable flue gas analyzer (Testo, Lenzkirch, Germany).

Exposure to BMF: The rats in the BMF group were exposed to smoke produced by smoldering China fir sawdust (30 g/exposure) for four 1-h periods, 5 days per week, for 4 weeks. The BMF was generated by a burn stove (500 w) for 10 min and the smoke was sent into the animal exposure room through a piston pump (5 L/min).

Exposure to MVE: A Wuyang model WY48QT-2, 1.6-Kw, 125-cm^3^, one-cylinder, four-cycle, gasoline-powered motorcycle (Guangzhou, China) was used as the MVE source. The motorcycle was operated using premium low-sulfur gasoline (<150 ppm; Petro Inc., El Paso, TX, USA) and 5 W-50 motor oil (ExxonMobil, Irving, TX, USA). The motorcycle engine was operated in an idle state for 2 min and then stopped for 10 min to achieve a stable mass concentration. The rats in the MVE group were exposed to PM for two 2-h exposure periods, 5 days per week, for 4 weeks.

### Sample preparation and isolation of alveolar macrophages (AMs)

Rats were sacrificed after the 4-week exposure period (day 29) using pentobarbitone (Sigma-Aldrich, St. Louis, MO, USA) administered by intra-peritoneal injection according to body weight (90 mg/kg). The lungs were cyclically inflated and deflated with 6 mL of phosphate-buffered saline (PBS) (Gibco-Thermo Fisher Scientific, Waltham, MA, USA). This was repeated for a total lavage fluid volume of 18 mL. For microbiome analysis, bronchoalveolar lavage fluid (BALF) was centrifuged at 15,000 *g* for 30 min at 4 °C. The supernatants were discarded and the pellets were snap-frozen in liquid nitrogen and stored at −80 °C. The remainder BALF was processed as described below.

BALF was centrifuged at 200 *g* for 5 min at 4 °C to obtain the cells and supernatants. The supernatant was frozen at −80 °C for biochemical analyses, and the cell pellet was rinsed and suspended in RPMI-1640 complete medium (Gibco-Thermo Fisher Scientific) with 10% fetal bovine serum (FBS) (Gibco-Thermo Fisher Scientific) and 1% penicillin/streptomycin (Gibco-Thermo Fisher Scientific). AMs were plated and incubated for 2 h at 37 °C in a humidified atmosphere of 5% CO_2_. Nonadherent cells were removed by gentle rinsing with complete medium.

### Differential BALF count

For Diff-Quik cell staining (Baso Diagnostics, Inc., Zhuhai, China), a portion of each sample was diluted to 2 × 10^4^ cells per 100 μL and fixed by cytospin (Shandon Inc., Pittsburgh, PA, USA). Differential cell counts were assessed based on 400 cells counted from each slide.

### Phagocytosis assay and confocal microscopy

Phagocytosis was measured as described previously [18, 19]. *Staphylococcus aureus* 1.2386 and *Streptococcus pneumoniae* 1.8722 were obtained from the China General Microbiological Culture Collection Center (Beijing, China) and fluorescently labeled using Alexa Fluor 488 dye (Thermo Fisher Scientific). Fluorescently-labelled polystyrene beads (2-μM diameter) were obtained from Thermo Fisher Scientific.

For the in vitro assay, AMs were plated onto 24-well plates (5 × 10^4^ cells/well; Corning, Corning, NY, USA) and incubated with *S. aureus* and *S. pneumoniae* at a ratio of 100 bacteria per cell for 2 h (fluorescent beads at a ratio of 50 beads per cell for 4 h) at 37 °C in a humidified atmosphere of 5% CO_2_. After incubation, cells were rinsed with Dulbecco’s PBS (D-PBS), and the fluorescence of extracellular bacteria was quenched by adding trypan blue (2%, *v*/v) for 1 min. AMs were rinsed again with D-PBS, trypsinized by trypsin (Gibco-Thermo Fisher Scientific), and resuspended in ice-cold PBS. A total of 10,000 cells were measured and analyzed using a BD Accuri® C6 flow cytometer (Becton Dickinson, Franklin Lakes, NJ, USA). Data were expressed as median fluorescence intensity (MFI).

Confocal microscopy was used to ascertain whether the beads were internalized as described previously [20]. Cells were viewed on a confocal microscope with a krypton-argon laser fluorescence detector (Zeiss LSM880, Oberkochen, Germany).

### Immunoglobulin assays

Immunoglobulin concentrations were measured in collected supernatants using commercial ELISA kits (Abcam, Cambridge, UK). Proteins measured included total IgA, IgG, and IgM. Briefly, each kit was a solid-phase sandwich ELISA utilizing monoclonal antibodies specific for the target protein. All procedures were performed according to the manufacturer’s instructions. Immunoglobulin concentrations were expressed as μg/mL of supernatant.

### 16S rRNA gene sequence analysis

Illumina sequencing was performed on amplicons from cDNA extracted from BALF samples of six BMF-exposed, six MVE-exposed, and six air-exposed rats. The total DNA from a 200-μL aliquot of the supernatant was extracted with the Qiagen DNA extraction kit (Qiagen, Hilden, Germany) according to the manufacturer’s recommendations. The V3–V4 regions of the 16S rRNA gene were amplified and sequenced on an Illumina MiSeq instrument (San Diego, CA, USA) using the 300 paired-end protocol at the Ribobo Genome Center (Guangzhou, China) as described previously [21].

Sequence reads were processed to remove low-quality reads. Unique reads were pre-clustered by Mothur software scripts [22]. Unique reads from each sample were clustered into a group of similar sequences, each representative of a 16 s rRNA sequence from a bacterial genus or species, and termed operational taxonomic units (OTUs). Next, OTU numbers were calculated using Mothur scripts based on 97% similarity. Unique reads were aligned to the Ribosome Database Project database [23] and annotated. OTUs were then classified to one species if more than 51% contained unique reads annotated to this species. QIIME software scripts (version 1.50) were used to analyze the distribution of all annotated species or OTUs in different taxonomic levels (phylum, class, order, family, genus) among different samples. Community (alpha) diversity indices combine species richness and abundance into a single value of evenness. As the most widely used indices, taxonomic alpha diversity was measured using OTU, Chao 1 indices, phylogenetic diversity whole tree (PD_whole_tree), and observed_species [24–26].

### Lung morphometric analysis

Lung tissues were fixed with 4% paraformaldehyde solution and embedded in paraffin using standard methods as described previously [27]. Sections (5-μm) were stained with hematoxylin and eosin (H&E) to detect cellular infiltration. For each animal, 10 fields were captured at a × 200 magnification in a blinded fashion using an image analyzer platform (Leica, Germany). Alveolar enlargement and destruction were quantified by the mean linear intercept (Lm), and the bronchial wall thickness was calculated as wall thickness = (total bronchial area − lumen area)/total bronchial area.

### Statistical analysis

Data are reported as mean ± standard error of the mean (SEM). Statistical analysis was performed in SPSS version 21 (IBM SPSS 21, Armonk, NY, USA) using Student’s *t*-test for normally distributed populations and the Mann–Whitney U-test for populations where normal distribution was not accomplished. A *p*-value of less than 0.05 was considered significant.

## Results

### Particle size distributions in suspension and gas concentrations in the exposure rooms during air pollution PM exposure

**Fig. 1:**
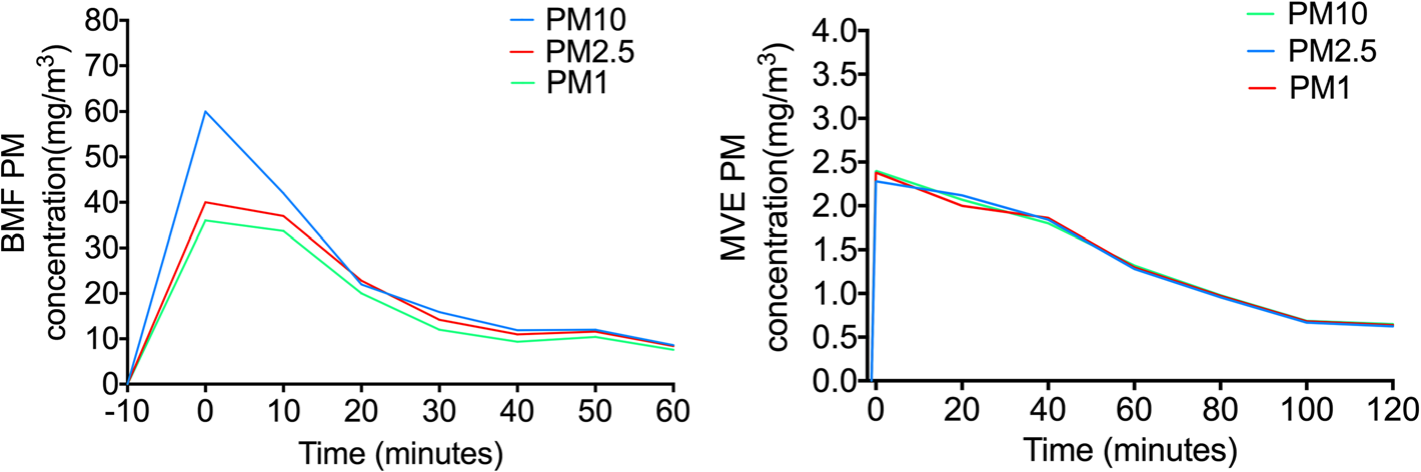
Particle size distribution during exposures to biomass fuel (BMF) and motor vehicle exhaust (MVE). Concentrations of PM_10_, PM_2.5_, and PM_1_ differed significantly in the BMF exposure room; PM_10_ concentrations were highest. Concentrations did not differ in the MVE exposure room. PM, particulate matter

PM mass concentrations and particle size distributions were measured during the PM exposures. The variations in both the BMF exposure room and the MVE exposure room are depicted in Fig. 1. We also measured gas concentrations inside the chambers, to estimate levels of gaseous co-pollutants generated by combustion. The mean ± SEM concentrations of various particle sizes (PM_10_, PM_2.5_, and PM_1_) and gases (O_2_, CO, NO_x,_and SO_2_) in the exposure rooms are shown in Table 1.

**Table 1:** Pollutant concentrations measured during exposures to biomass fuel (BMF) and motor vehicle exhaust (MVE)

	CON	BMF	MVE
PM10 (mg/m^3^)	0.076 ± 0.007	24.6 ± 7.3	1.40 ± 0.26
PM2.5 (mg/m^3^)	0.076 ± 0.007	20.7 ± 4.9	1.40 ± 0.26
PM1 (mg/m^3^)	0.076 ± 0.007	18.4 ± 4.5	1.41 ± 0.26
NO_1_ (ppm)	—	0.64 ± 0.067	0.23 ± 0.04
NO_X_ (ppm)	—	0.64 ± 0.067	0.23 ± 0.04
SO_2_ (ppm)	—	—	0.72 ± 0.07
CO (ppm)	—	95.6 ± 4.2	73.6 ± 3.7
O_2_ (%)	20.9 ± 0.01	20.83 ± 0.04	20.92 ± 0.03
Humidity (%)	57.4 ± 2.9	59.3 ± 2.3	62.6 ± 5.3
Temperature (°C)	24.3 ± 0.2	25.76 ± 0.31	24.8 ± 0.3

Values are shown as mean ± SEM. CON, control group; BMF, biomass fuel exposure group; MVE, motor vehicle exhaust exposure group; PM, particulate matter

### Phagocytosis by AMs following PM exposure

**Fig. 2:**
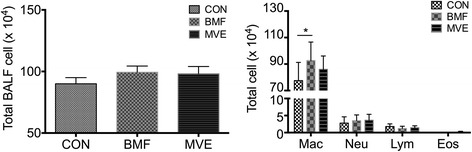
Percentages of macrophages in bronchoalveolar lavage fluid (BALF). Following particulate matter exposure, the percentage of macrophages increased in BALF from rats exposed to biomass fuel (BMF). Results are expressed as mean ± SEM; *n* = 8 rats. CON, control; MVE, motor vehicle exhaust; Mac, macrophages; Neu, neutrophils; Lym, lymphocytes; Eos, eosinophils. *p < 0.05

There were no significant differences in total BALF cell counts between the PM exposure group and control group, but the BALF macrophage counts increased in the BMF group compared with the control group (*p* = 0.045) (Fig. 2).

**Fig. 3:**
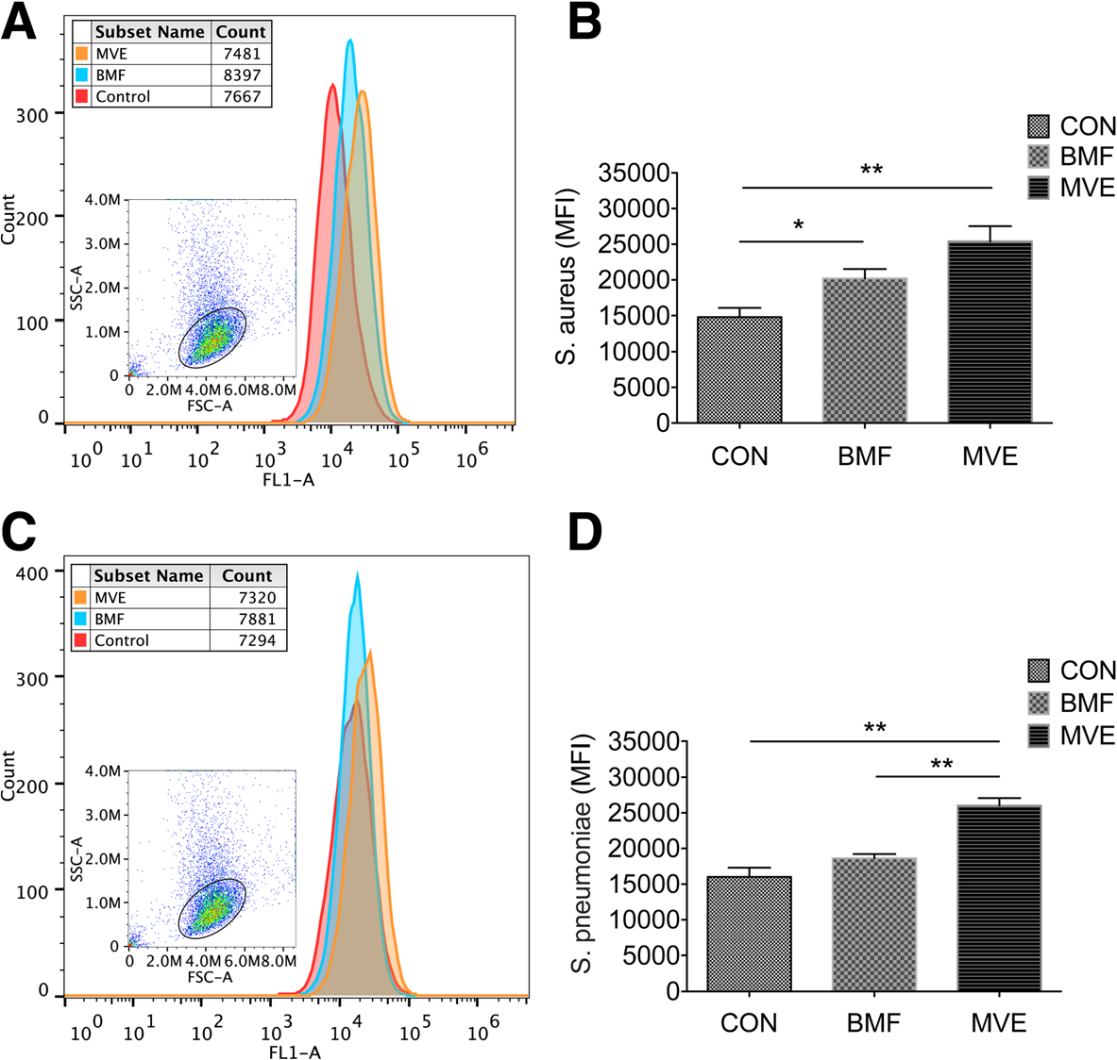
The phagocytic response of alveolar macrophages (AMs) to fluorescently labeled bacteria following a 4-week exposure. **a**, **b** AM capacity to phagocytose *Staphylococcus aureus* increased significantly following exposure to particulate matter (PM), especially PM from motor vehicle exhaust (MVE) emissions. **c**, **d** AMs from rats exposed to MVE exhibited an increased capacity to ingest *Streptococcus pneumoniae* compared with AMs from air-exposed and biomass fuel (BMF)-exposed rats. Results are expressed as mean ± SEM; *n* = 8 rats. CON, control group; MFI, median fluorescence intensity. *p < 0.05, **p < 0.01

**Fig. 4:**
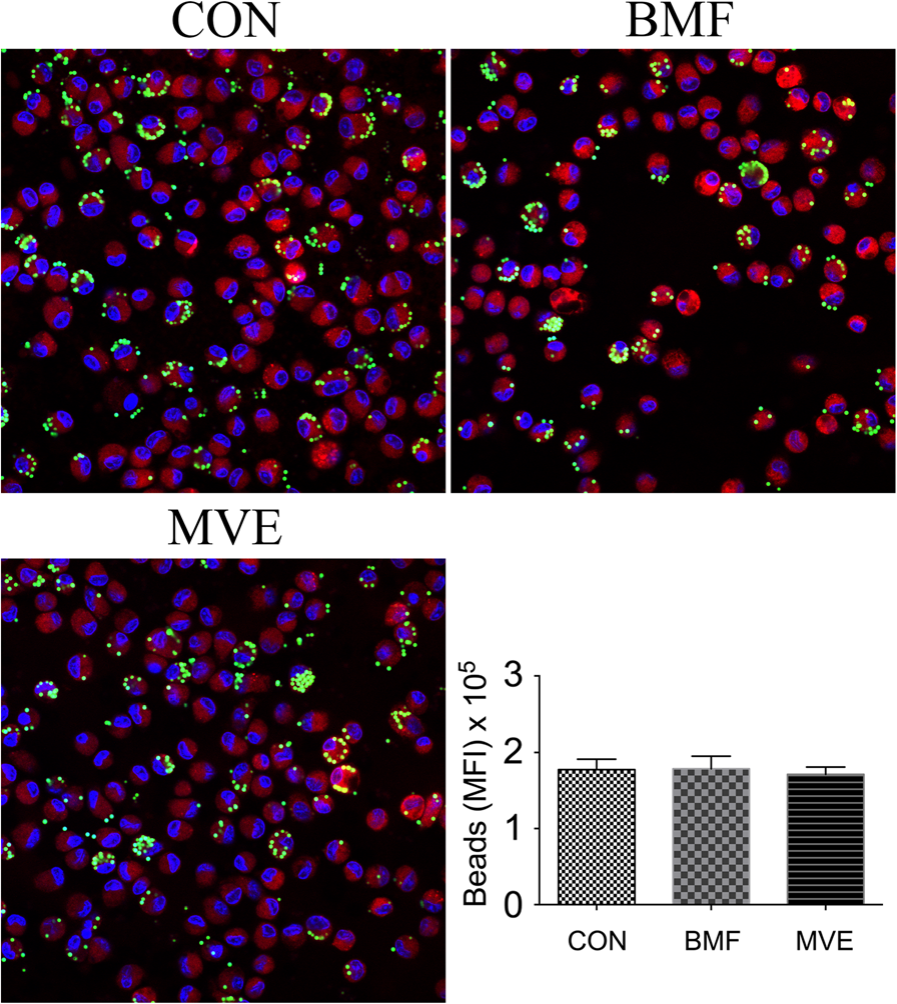
Confocal microscopy and phagocytosis of fluorescently-labeled beads by alveolar macrophages. There was no difference in phagocytosis of beads between the three groups. Internalization of beads was confirmed using confocal microscopy. Results are expressed as mean ± SEM; *n* = 4 rats. CON, control group; BMF, biomass fuel exposure group; MVE, motor vehicle exhaust exposure group. *p < 0.05, **p < 0.01

The phagocytic response of AMs to fluorescently labeled bacteria or beads was determined by flow cytometry. AMs from the three groups exhibited differential capacities to phagocytose bacteria. Increased phagocytosis of *S. aureus* and *S. pneumoniae* was observed in the MVE exposure group, compared with the control group (*p* < 0.01). AMs from the BMF-exposed group exhibited an increased capacity to ingest *S. aureus* (*p* = 0.012), but did not differ from AMs derived from control rats in their capacity to ingest *S. pneumoniae* (*p* = 0.097) (Fig. 3). However, there was no difference in phagocytosis of beads between the three groups (Fig. 4).

### PM exposure induces immunoglobulin changes

**Fig. 5:**
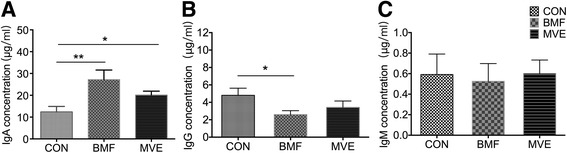
Immunoglobulin levels in bronchoalveolar lavage fluid following a 4-week exposure to particulate matter. (**a**) Levels of IgA. **b** Levels of IgG. **c** Levels of IgM. Results are expressed as mean ± SEM; *n* = 8 rats. CON, control group; BMF, biomass fuel exposure group; MVE, motor vehicle exhaust exposure group. *p < 0.05, **p < 0.01

Concentrations of total IgA, IgG, and IgM were measured using ELISA kits. IgA levels in BALF increased following the 4-week exposure to BMF (*p* < 0.01) and MVE (*p* = 0.02) compared with air exposure (Fig. 5a). In contrast, IgG levels decreased significantly in the BMF group compared with controls (*p* = 0.031). A downward trend that was not statistically significant was seen in the MVE group (Fig. 5b). BALF from all groups contained similar concentrations of IgM (Fig. 5c). Immunoglobulin concentrations were expressed as μg/mL of the BALF supernatant.

### Effects of PM exposure on lung morphology

**Fig. 6:**
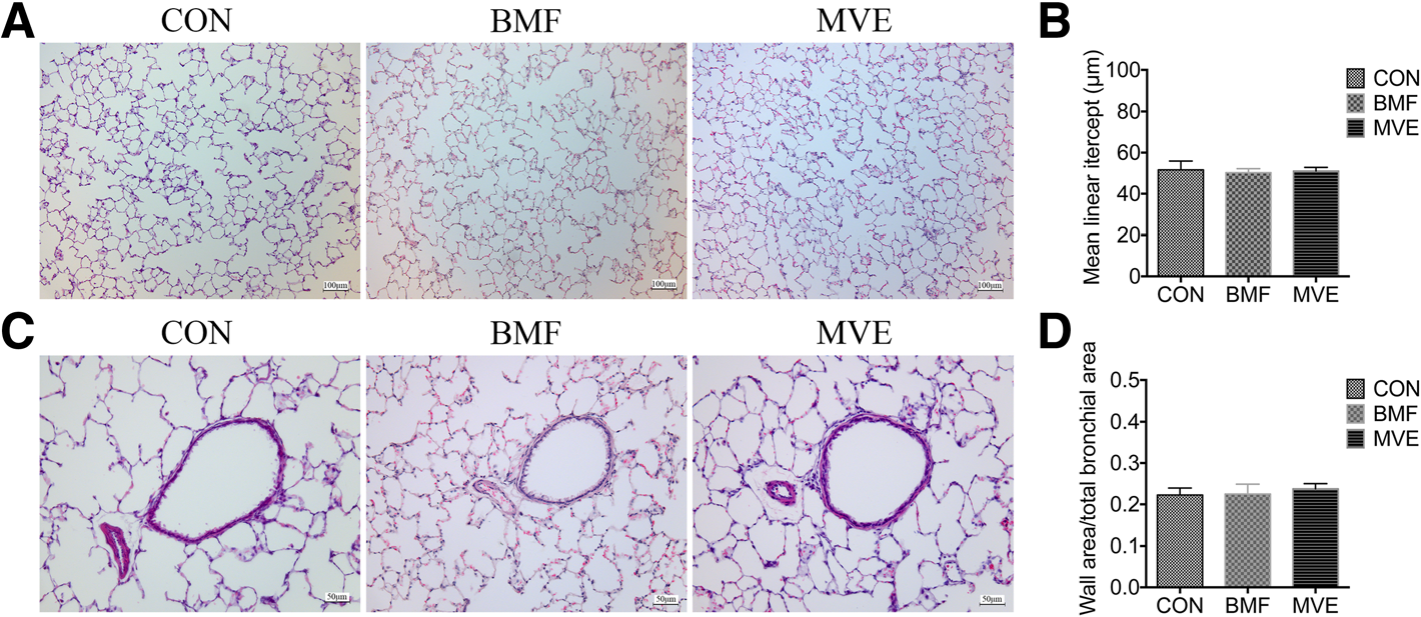
Lung morphological changes following a 4-week exposure to particulate matter. (**a** and **c**) Representative images of lung sections from rats exposed to clean air (CON), biomass fuel (BMF), and motor vehicle exhaust (MVE). **b** and **d** Changes in mean linear intercepts and ratio of wall area to total bronchial area in lung sections from rats exposed to CON, BMF, and MVE. Results are expressed as mean ± SEM; *n* = 5 rats. No significant changes were observed between groups

Figure 6 (a, b) shows that the Lm values in the lungs of rats exposed to BMF and MVE for 4 weeks did not differ from those of rats exposed to clean air. Moreover, the thickness of bronchial walls in the lungs of rats exposed to BMF and MVE particles was similar to that of control rats (Fig. 6c, d), suggesting that the exposures did not induce morphological changes.

### Changes in lung bacterial diversity following PM exposure

**Fig. 7:**
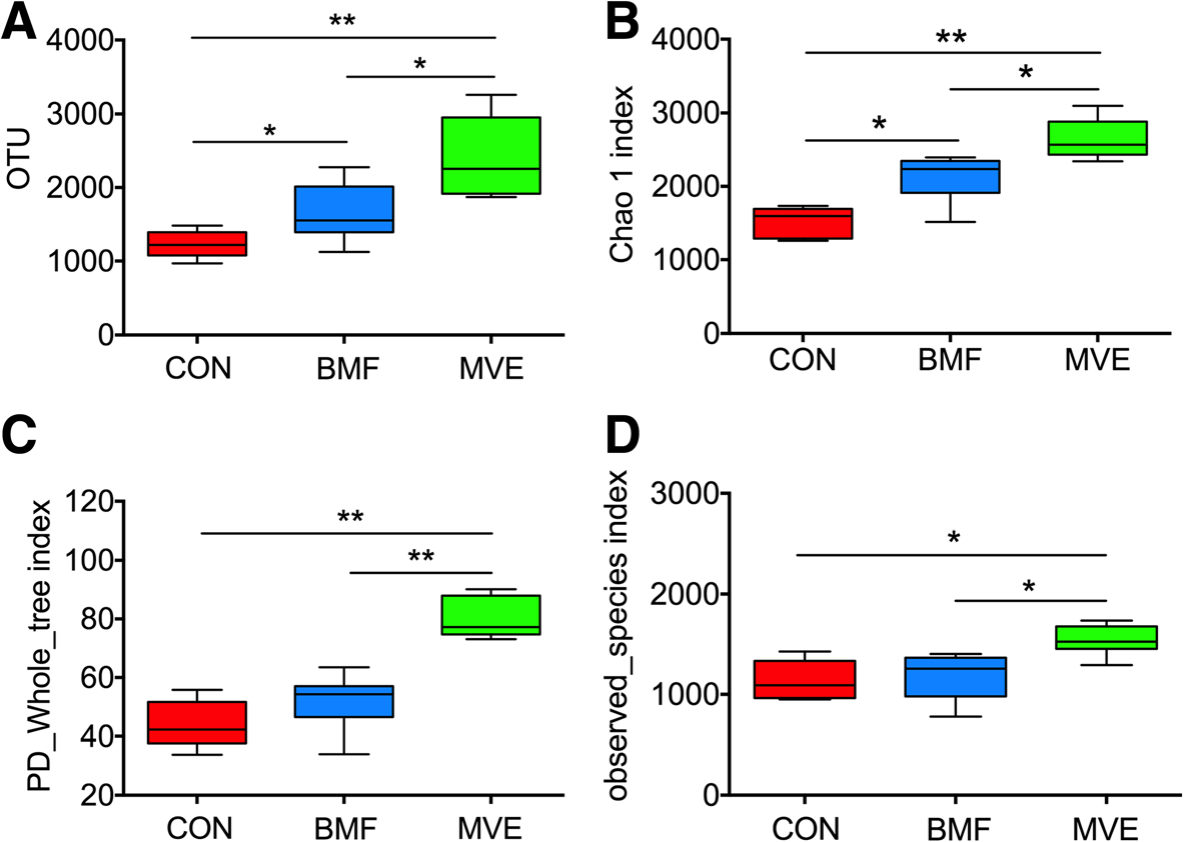
Microbial abundance and diversity in exposure groups. Boxplots of alpha diversity measures of species richness (operational taxonomic unit [OTU] number, **a**) and microbial diversity (Chao 1 index, PD_whole_tree index, and observed_species index, **b**–**d**). Results are expressed as mean ± SEM; *n* = 6 rats. CON, control group; BMF, biomass fuel exposure group; MVE, motor vehicle exhaust exposure group. * *p* < 0.05, ** *p* < 0.01

A total of 1,484,863 raw reads were generated for all samples, with an average of 82,492 total reads per sample. After filtering for low-quality reads, 1,205,883 sequence reads were used for subsequent analyses and resulted in 31,701 OTUs (Table 2). Comparison between the three groups showed that bacterial abundance (OTU number) increased significantly in the BMF group and MVE group compared with the control group (*p* = 0.021 and *p* < 0.01, respectively). The mean (SEM) OTU numbers for the control, BMF, and MVE groups were 1227.8 (78.6), 1651.8 (162.8) and 2403.8 (221.9), respectively (Fig. 7a). The MVE group exhibited higher bacterial diversity than the BMF and control groups; the BMF group exhibited higher bacterial diversity than the control group (Fig. 7b–d).

**Table 2:** Sequence characteristics of samples from the control, biomass fuel, and motor vehicle exhaust groups

Sample	Raw reads	Clean reads	Clean rate (%)	OTUs
C1	48,881	47,021	95.4	1482
C2	42,486	40,658	94.9	968
C3	51,592	48,734	93.6	1363
C4	61,056	58,690	95.2	1115
C5	84,335	64,554	67.2	1114
C6	64,101	61,549	95.3	1325
B1	99,701	73,766	71.7	2277
B2	96,756	73,463	70.1	1926
B3	106,549	82,315	68.7	1481
B4	101,385	78,044	68.0	1560
B5	103,303	76,117	65.0	1544
B6	82,009	65,377	72.8	1123
M1	83,505	69,310	77.3	1933
M2	69,562	59,557	83.1	3254
M3	110,918	92,134	77.8	2855
M4	67,860	57,459	76.2	1870
M5	102,845	76,430	67.4	2266
M6	108,019	80,705	66.8	2245

C1–C6, control group; B1–B6, biomass fuel exposure group; M1–M6, motor vehicle exhaust exposure group; *n* = 6 experiments. OUT, operational taxonomic unit

### Lung bacterial composition

**Fig. 8:**
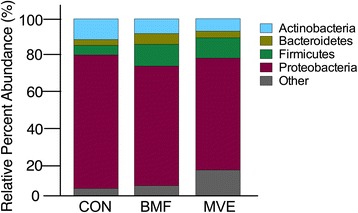
Phylum percent relative abundances. Microbial abundance was measured in bronchoalveolar lavage fluid from rats exposed to clean air (CON) and to particulate matter from biomass fuel (BMF) and motor vehicle exhaust (MVE). The phylum distribution differed significantly between the three groups, driven by an increase in *Proteobacteria*

To determine the response of the host microbiome to PM, we analyzed the taxonomical community structure of the microbiome in BALF samples of air-exposed, BMF-exposed, and MVE-exposed rats. At the phylum level, all samples from control, BMF, and MVE groups contained four major bacterial phyla (%): *Proteobacteria*, 75.5 ± 1.5, 67.5 ± 8.1, and 62.6 ± 4.5, respectively; *Firmicutes*, 5.4 ± 0.9, 12.3 ± 5.6, and 11.4 ± 2.1, respectively; *Bacteroidetes*; 3.3 ± 0.4, 6.1 ± 2.7, and 3.7 ± 0.8, respectively; *Actinobacteria*, 11.7 ± 1.8, 8.3 ± 3.9, and 7.0 ± 1.1 respectively. The first three phyla accounted for over 80% of the total sequences in all three groups (Fig. 8). Relative abundances of bacterial phyla differed (*p* < 0.05) between the control and MVE groups. On the basis of Bonferroni post hoc testing, expansion of the *Proteobacteria* phylum was the most significant driver of the difference between the control group and the MVE group (75.5 ± 1.5 vs. 62.6 ± 4.5, *p* = 0.017). There was no significant difference between the control group and BMF group.

**Fig. 9:**
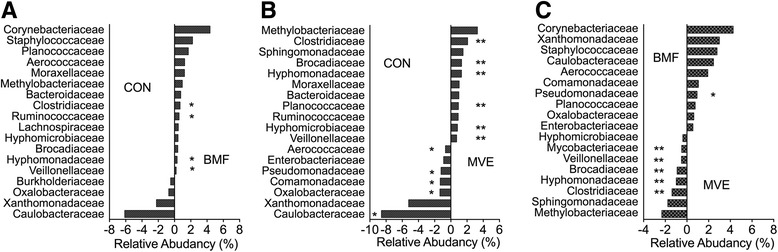
Relative abundance of 20 bacterial families. The abundance of major bacterial families was measured in samples comparing bronchoalveolar lavage fluid from rats exposed to (**a**) clean air (CON) and biomass fuel (BMF), (**b**) CON and motor vehicle exhaust (MVE), and (**c**) BMF and MVE. The value of zero indicates a similar percentage contribution of the bacterial family in any two groups. A positive value for a bacterial family indicates higher percent relative abundance in the group on the right, and a negative value indicates higher abundance in the group on the left. * *p* < 0.05, ** *p* < 0.01

At the family level, on the basis of these assignments, the sequences represented in the four phyla were distributed into 67 bacterial families. Twelve bacterial families differed in their relative abundance in samples from the three groups (Fig. 9). *Clostridiaceae*, *Ruminococcaceae*, *Hyphomonadaceae*, and *Veillonellaceae* were present in higher proportions in the BMF group than in the control group (Fig. 9a). *Clostridiaceae*, *Brocadiaceae*, *Hyphomonadaceae*, *Planococcaceae*, *Hyphomicrobiaceae*, and *Veillonellaceae* were present in higher proportions in the MVE group, whereas *Aerococcaceae*, *Pseudomonadaceae*, *Comamonadaceae*, *Oxalobacteraceae*, and *Caulobacteraceae* were present in lower proportions than in the control group (Fig. 9b). A comparison of the BMF and MVE groups showed a higher prevalence of *Clostridiaceae*, *Hyphomonadaceae*, *Brocadiaceae*, *Veillonellaceae*, and *Mycobacteriaceae* in the BMF group, while *Pseudomonadaceae* were more prevalent in the MVE group (Fig. 9c).

## Discussion

Air pollution is a major environmental issue contributing to chronic respiratory diseases; industry, traffic, and household biomass combustion have become major sources of ambient PM emissions [3]. Moreover, incomplete combustion of biomass fuel and living in proximity to traffic have been associated with a high prevalence of chronic obstructive pulmonary disease (COPD) [28, 29], which is why we elected to focus on BMF and MVE in our study. The PM concentrations of BMF and MVE reported here are in accordance with those in our previous studies, which measured the upper bound of indoor air pollution generated during cooking in rural areas of Southern China [5, 29, 30], and with those measured in Northern China under heavy haze conditions [31]. Exposure to ambient particulate matter for 6 months causes airway cells to release multiple cytokines capable of inducing pronounced COPD in rat models [10]. We therefore selected a short-term exposure to determine whether PM alters the lung microbial composition before lung morphometric changes occur. In this study, we demonstrated that a 4-week exposure of rats to airborne PM increased the lung bacterial load and diversity, and altered the microbial composition before any histopathological changes occurred. Furthermore, PM exposure increased phagocytosis by AMs and induced changes in immunoglobulins.

Microdysbiosis is defined as quantitative and qualitative changes in the microbiota, including an increase in common bacterial inhabitants that can become pathogenic under selective pressures [32]. Previous studies support the hypothesis that inhaled air pollution may impact the microbial composition of the lung [33, 34]. These studies found that exposure to higher levels of particulates resulted in higher abundances of potentially pathogenic bacteria within the lung microbiome. We found that samples from the BMF and MVE groups contained higher bacterial abundances and bacterial diversity than samples from the control group, based on the OTU numbers and alpha diversity, supporting the study of Yu and colleagues, who demonstrated that microbiota taxonomic alpha diversity increases with environmental exposures, and that specific aspects of the lung microbiota are related to different sources of PM [33]. Rylance and colleagues reported that domestic BMF use was associated with an uncommon environmental bacterium (*Petrobacter*) usually found in oil reservoirs [34]. We found that the distribution of phyla in the MVE group differed significantly from distribution in the control and BMF groups (Fig. 8), and that this change was driven by increases in *Proteobacteria*. At the family level, 12 bacterial families differed in their relative abundance in samples from the three groups (Fig. 9), not only between the PM exposure groups and the controls, but also within the exposure groups. *Clostridiaceae*, *Hyphomonadaceae*, *Brocadiaceae*, *Veillonellaceae*, and *Mycobacteriaceae* were more prevalent in the BMF group, whereas *Pseudomonadaceae* numbers were higher in the MVE group (Fig. 9c).

We also compared the histopathological changes in the lungs of rats exposed to BMF or MVE, but found no significant difference in bronchial wall thickness or alveolar enlargement (Fig. 6). Combined with our previous findings from a 6-month exposure [10], the results indicate that BMF and MVE exposure alters the lung microbiome before lung morphometric changes occur. Moreover, some alterations in bacterial composition were found in the lungs of rats exposed to BMF or MVE, supporting the hypothesis that BMF and MVE exposure can result in various COPD phenotypes [35].

Exposure to ambient PM also affects innate and adaptive immune responses in the rat lung, and this may be partly explained by disordered microbial communities. AMs are known as the principal cells that process airborne particles in the lung and act as antigen-presenting cells to activate the adaptive immune response [36]. We found that BALF macrophage counts increased in the BMF group. This is in agreement with previous studies that reported an increase in phagocytic inflammatory cell numbers in BALF following exposure to air pollution [37]. Exposure to PM stimulates the bone marrow, promoting the release of monocytes and their recruitment into the lung, where they subsequently differentiate into tissue and alveolar macrophages [38]. In our study, AMs from the BMF and MVE exposure groups displayed an increased capacity to phagocytose bacteria. However, the phagocytic response of these macrophages to polystyrene beads was not altered in cells from the BMF and MVE exposure groups, indicating that the change was related specifically to these bacteria. This finding is distinct from that of previous studies [39, 40], which reported that PM exposures significantly reduced the uptake of pathogens. However, our results were obtained from in vivo experiments, and high-level PM exposures in vitro may not represent biological conditions in an ambient air-polluted environment [41]. Macrophage phagocytic activity increases with increasing PM concentrations, but will be impaired when the phagocytic particles exceed 60% of the intracellular volume, overloading the macrophages [42, 43].

Few data are available on the effects of ambient PM on immune functions in the lung. Some work on PM has centered on alterations in immunoglobulin production [44]. We found that PM exposure increased total IgA levels in BALF samples from both the BMF and MVE exposure groups (Fig. 5a), similarly to the findings of Gaschler and colleagues, who reported that an 8-week exposure to cigarette smoke increased IgA titers in BALF from mice [45]. Interestingly, PM exposure in our study decreased total IgG levels in the BMF group and did not significantly alter concentrations of IgM in the three groups (Fig. 5b, c). Basilico and colleagues reported that total IgA and IgG levels in mice exposed to cigarette smoke for 6 weeks were similar to or lower than levels in controls [46]. Several experimental factors may explain the discordant findings: Different exposures (our study did not include cigarettes), the duration and intensity of the exposure, and the animal species and strain used. In general, our study and the published literature show alterations in the adaptive immune response in rodent lungs following a PM exposure.

In summary, we demonstrated that a short-term PM exposure can alter the microbial composition and affect innate and adaptive immune responses in the rat lung. Furthermore, defining the composition of the resident microbiome and how microbial communities shift with exposure may help to explain their role in responding to inhaled particulate matter.

## Conclusion

This study found that a short-term PM exposure can alter the microbial composition and induce immune changes in the rat lung.

## Abbreviations

AM: Alveolar macrophages
BALF: Bronchoalveolar lavage fluid
BMF: Biomass fuel
MFI: Median fluorescence intensity
MVE: Motor vehicle exhaust
OTU: Operational taxonomic unit
PM: Particulate matter
